# Supplementary material for: Effect of Bacterial Amyloid Protein Phenol−Soluble Modulin Alpha 3 on the Aggregation of Amyloid Beta Protein Associated with Alzheimer’s Disease
Source: Biomimetics (Basel). 2023 Oct 1;8(6):459. doi: 10.3390/biomimetics8060459 (PMC10604207; doi:10.3390/biomimetics8060459)
Supplement: Supplementary file 1 [file biomimetics-08-00459-s001.zip › biomimetics-2559199-supplementary.pdf]

Supplementary Information

Effect of Bacterial Amyloid Protein Phenol−Soluble Modulin Alpha 3 on the Aggregation of Amyloid Beta Protein Associated with Alzheimer's Disease

Bushu Peng, Shaoying Xu, Yue Liang, Xiaoyan Dong *, Yan Sun *

Department of Biochemical Engineering, School of Chemical Engineering and Technology, Key Laboratory of Systems Bioengineering and Frontiers Science Center for Synthetic Biology (Ministry of Education), Tianjin University, Tianjin 300350, China.

CONTENTS

Table S1 − Table S8 ·································· S2−S9

Figure S1 − Figure S7 ································· S10−S16

Table S1. Maximum ThT fluorescence intensity (y_{max}) and lag phase time (T_{lag}) of aggregation kinetics of Aβ_{40} (25 μM) with different concentrations of PSMα3 monomers. The standard errors of the mean were calculated from three repeats.

**Aβ_{40} : PSMα3**	**y_{max} (a.u.)**	**T_{lag} (h)**
1 : 0	13162.2 ± 470.6	52.8 ± 1.0
1 : 0.05	9863.1 ± 182.5	56.6 ± 0.9
1 : 0.1	8071.9 ± 516.8	62.3 ± 2.2
1 : 0.2	4171.4 ± 87.1	64.7 ± 1.3
1 : 0.5	1499.3 ± 210.9	77.1 ± 6.3
1 : 1	1027.2 ± 44.2	92.1 ± 1.2

Table S2. Maximum ThT fluorescence intensity (y_{max}) and lag phase time (T_{lag}) of aggregation kinetics of Aβ_{40} (25 μM) incubated without or with PSMα3 seeds. The standard errors of the mean were calculated from three repeats.

**Sample**	$\mathbf{y_{max}}$ **(a.u.)**	$\mathbf{T_{lag}}$ **(h)**
Aβ_{40}	13162.2 ± 470.6	52.8 ± 1.0
with PSMα3 seeds at 1 h	12700.0 ± 585.0	39.1 ± 2.3
with PSMα3 seeds at 2.5 h	14410.4 ± 644.1	33.8 ± 2.6
with PSMα3 seeds at 5 h	14311.0 ± 513.0	44.4 ± 1.8
with PSMα3 seeds at 24 h	13958.4 ± 459.0	47.6 ± 1.4

Table S3. Top 10 binding energies of the PSMα3m−$A\beta_{40}$ complex calculated by molecular docking using Autodock Vina [1].

Mode	Binding energy (kcal/mol)
1	−6.2
2	−5.9
3	−5.3
4	−5.2
5	−5.1
6	−5.1
7	−5.0
8	−4.9
9	−4.6
10	−4.5

Table S4. Top 10 binding energies of PSMα3o and $A\beta_{40}$ calculated by molecular docking using Autodock Vina [1].

Mode	Binding energy (kcal/mol)
1	−13.0
2	−12.7
3	−12.7
4	−12.1
5	−11.6
6	−11.5
7	−10.9
8	−10.7
9	−10.7
10	−10.5

Table S5. Structural parameters of the Aβ_{40} monomer in different systems in the equilibrium state. The standard errors of the mean were calculated from three repeats.

System	R_g [a] (nm)	SASA [b] (nm^2)	H−bond [c]
Aβ_{40} only	1.1 ± 0.03	38.5 ± 1.1	22
Aβ_{40}−PSMα3m	1.3 ± 0.03	40.3 ± 1.7	20
Aβ_{40}−PSMα3o	1.3 ± 0.04	42.1 ± 1.0	19

[a] Radius of gyration.

[b] Solvent accessible surface area.

[c] Number of intermolecular hydrogen bonds.

Table S6. Secondary structure composition of the Aβ_{40} monomer in different systems in the equilibrium state.

System (%)	Coil	β−bridge	Bend	Turn	α−helix
Aβ_{40} only	43.9	4.5	13.2	24.6	13.8
Aβ_{40}−PSMα3m	29.1	0.0	10.5	24.1	36.3
Aβ_{40}−PSMα3o	32.5	0.5	11.2	23.3	32.5

Table S7. Binding free energy components of the $A\beta_{40}$−PSMα3m and $A\beta_{40}$−PSMα3o systems calculated using the MM−PBSA method [2].

System (kJ/mol)	ΔG_{vdw} [a]	ΔG_{elec} [b]	ΔG_{ps} [c]	ΔG_{nps} [d]	ΔG_{bind} [e]
$A\beta_{40}$−PSMα3m	−373.5	−963.9	1106.5	−39.8	−270.7
$A\beta_{40}$−PSMα3o	−251.0	−1274.7	1418.9	−28.6	−135.5

[a] ΔG_{vdw} represents the van der Waals hydrophobic energy.

[b] ΔG_{elec} represents electrostatic energy under vacuum.

[c] ΔG_{ps} represents polar solvation energy.

[d] ΔG_{nps} represents nonpolar solvation energy.

[e] ΔG_{bind} represents total binding free energy.

Table S8. Energy components of key residues of Aβ40−PSMα3m system.

System (kJ/mol)	Residues	ΔG_{MM} [a]	ΔG_{ps} [b]	ΔG_{nps} [c]	ΔG_{TOT} [d]
Aβ40	ASP−1	−136.7	118.9	−1.9	−19.7
	LEU−17	−10.8	−3.1	−2.2	−16.1
PSMα3m	MET−1	−269.0	241.3	−2.2	−29.9
	GLU−2	107.7	−128.9	−0.5	−21.7
	PHE−3	−35.8	14.6	−2.3	−23.5
	LEU−7	−24.9	5.1	−2.6	−22.5
	LEU−14	−30.5	9.5	−2.5	−23.5
	PHE−18	−23.6	7.3	−1.7	−18.0

[a] $\Delta G_{MM} = \Delta G_{elec} + \Delta G_{vdw}$, vacuum energy.

[b] ΔG_{ps} represents polar solvation energy.

[c] ΔG_{nps} represents nonpolar solvation energy.

[d] ΔG_{TOT} represents total contribution of residues to binding free energy.

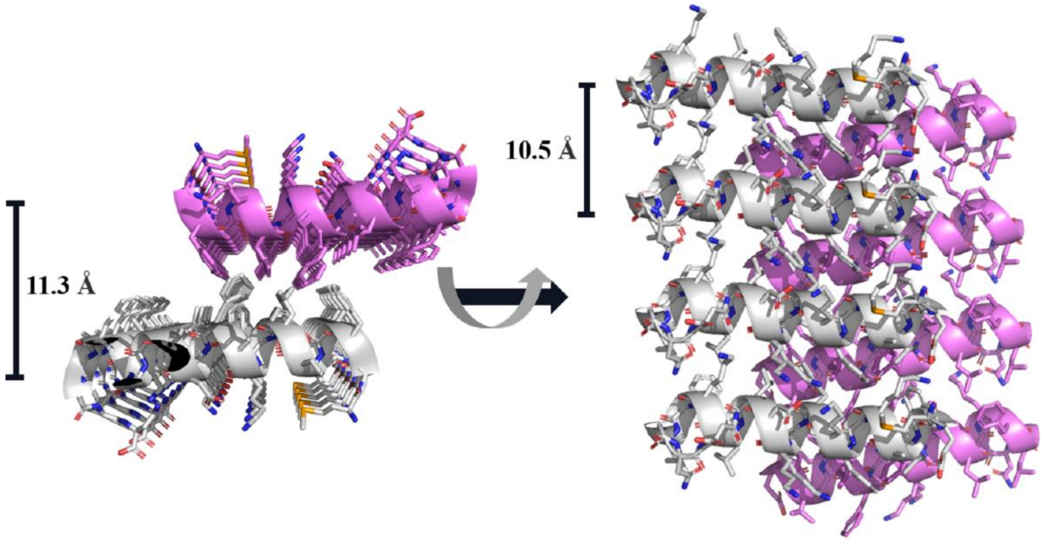

Figure S1. Crystal structure of PSMα3 (PBD ID: 5I55) [3].

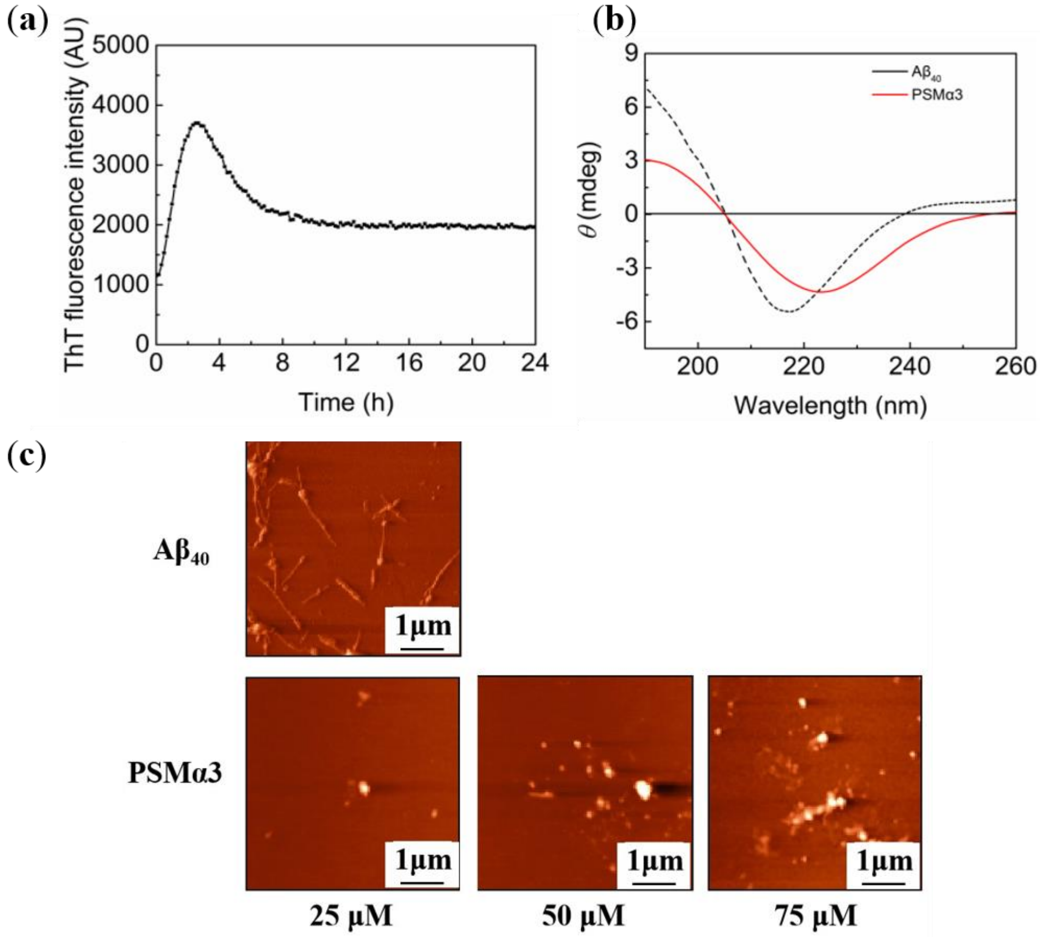

Figure S2. Aggregation kinetics of PSMα3: (**a**) Aggregation kinetics of PSMα3 (25 μM) by ThT fluorescence assays; (**b**) Far−UV circular dichroism spectra of $A\beta_{40}$ (25 μM) and PSMα3 (25 μM) aggregates at 72 h; (**c**) AFM images of $A\beta_{40}$ (25 μM) and PSMα3 (25 μM, 50 μM, 75 μM) aggregates obtained by incubation at 37 °C for 72 h.

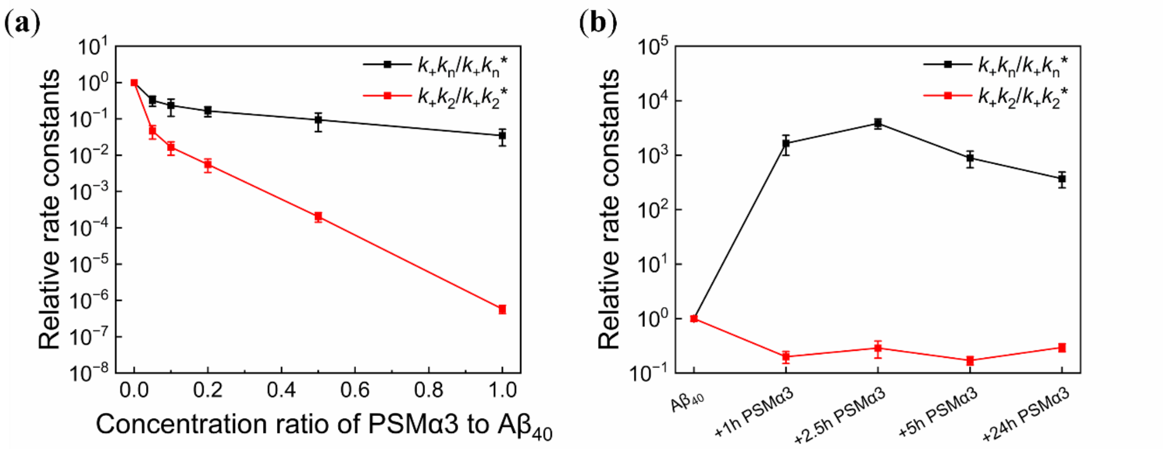

Figure S3. Relative kinetic rate constants of Aβ40 aggregation in the presence of (**a**) PSMα3 monomers or (**b**) PSMα3 seeds. The kinetic curve was fitted using the multistep secondary nucleation dominated, unseeded model with both k_nk_+ and k_2k_+ as free−fitting parameters [4]. An asterisk (*) denotes rate constants from Aβ40 aggregation in the absence of PSMα3. Error bars are standard deviations (s.d.) calculated from three repeats.

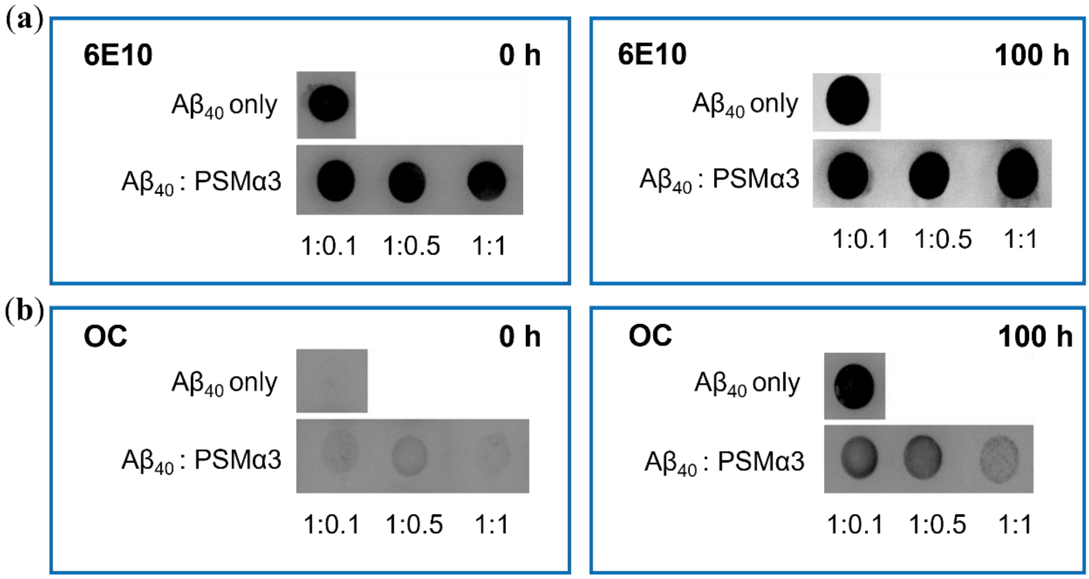

Figure S4. Effects of PSMα3 monomers at different concentrations on the formation of Aβ_{40} (25 μM) fibrils at 0 h and 100 h. (**a**) Aβ_{1-16} sequence–specific 6E10 antibody assay for all forms of Aβ species; (**b**) Aβ fibril–specific OC antibody assay.

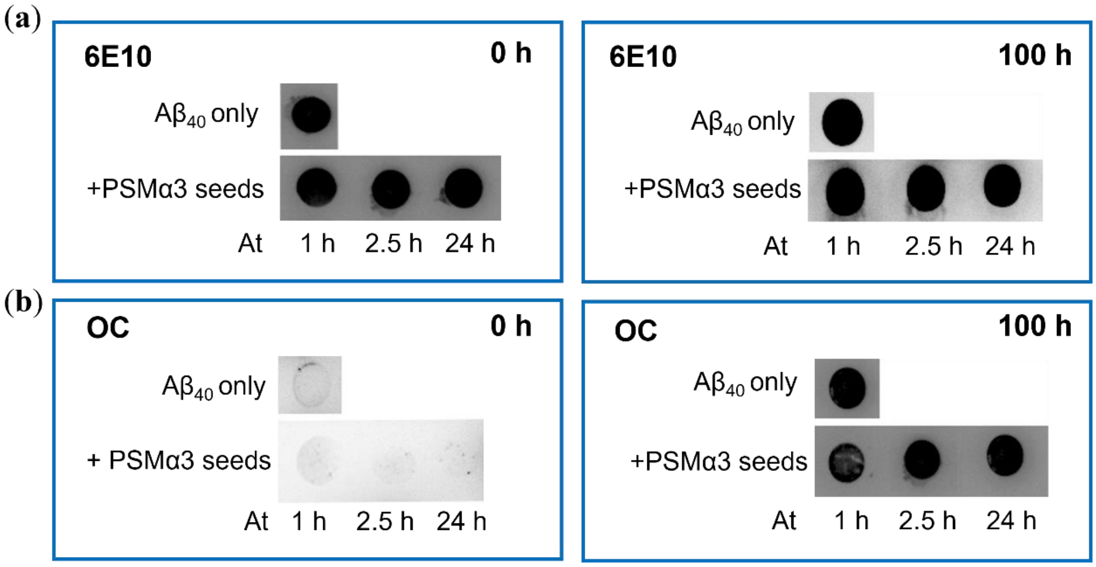

Figure S5. Effects of PSMα3 seeds (5 μM) at 1 h, 2.5 h, and 24 h on the formation of Aβ_{40} (25 μM) fibrils at 0 h and 100 h. (**a**) Aβ_{1-16} sequence−specific 6E10 antibody assay for all forms of Aβ species; (**b**) Aβ fibril−specific OC antibody assay.

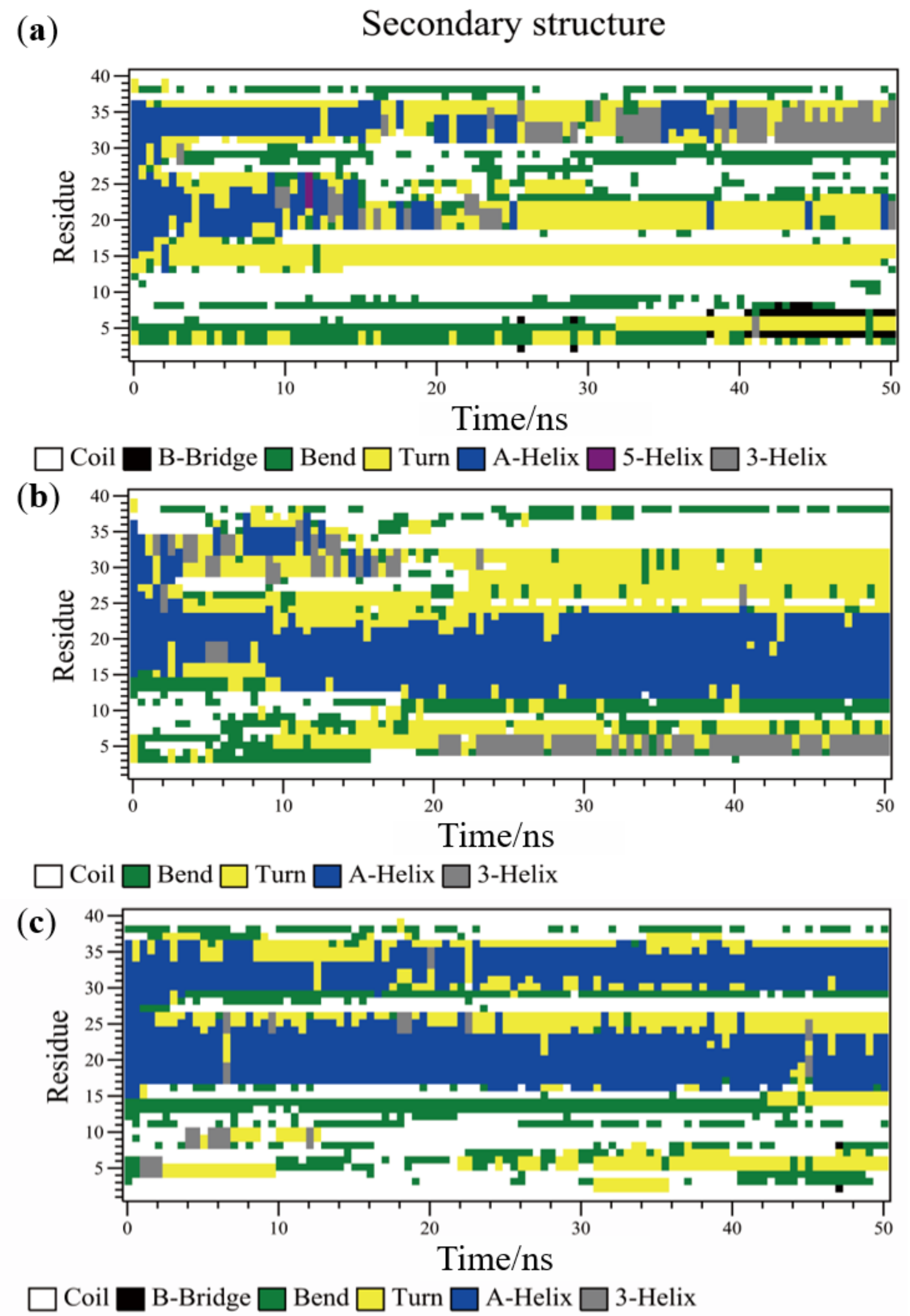

Figure S6. The secondary structure components of Aβ40 during the 50 ns MD simulations in (**a**) Aβ40 only, (**b**) Aβ40−PSMα3m, and (**c**) Aβ40−PSMα3o systems.

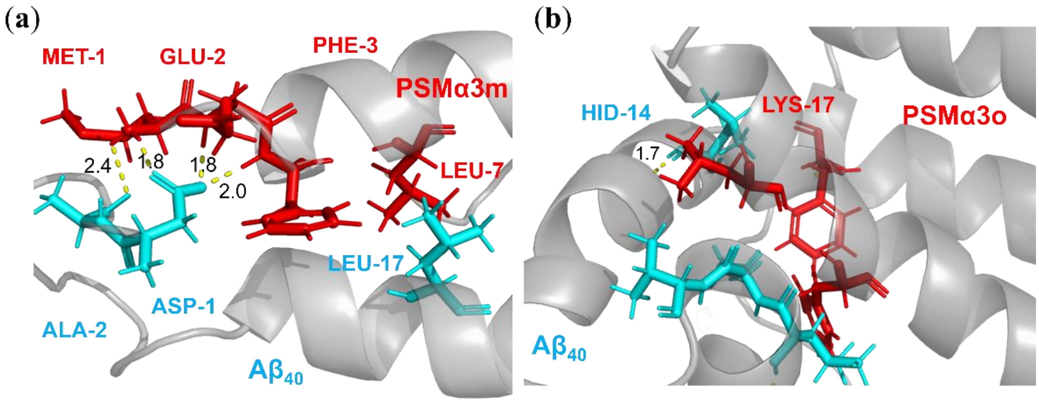

Figure S7. H−bonds between the Aβ_{40} monomer and (**a**) PSMα3m or (**b**) PSMα3o. Blue represents residues of Aβ_{40}, red represents residues of PSMα3m and PSMα3o.

References

(1) Trott, O.; Olson, A.J. Software news and update AutoDock Vina: improving the speed and accuracy of docking with a new scoring function, efficient optimization, and multithreading. *Comput Chem.* **2010**, *31* (2), 455−461.

(2) Kumari, R.; Kumar, R.; Lynn, A. g_mmpbsa−a GROMACS tool for high−throughput MM−PBSA calculations. *J. Chem. Inf. Model.* **2014**, *54* (7), 1951−1962.

(3) Tayeb−Fligelman, E.; Tabachnikov, O.; Moshe, A.; Goldshmidt−Tran, O.; Sawaya, M.R.; Coquelle, N.; Colletier, J.P.; Landau, M. The cytotoxic Staphylococcus aureus PSMα3 reveals a cross−α amyloid−like fibril. *Science* **2017**, *355* (6327), 831−833.

(4) Belsare K.D.; Wu H.; Mondal D.; Bond A.; Castillo E.; Jin J.; Jo H.; Roush A.E.; Pilla K.B.; Sali A.; et al. Soluble TREM2 inhibits secondary nucleation of Aβ fibrillization and enhances cellular uptake of fibrillar Aβ. *Proc. Natl. Acad. Sci. U. S. A.* **2022**, *119* (5), e2114486119.
